# Development of doubled haploid inducer lines facilitates selection of superior haploid inducers in maize

**DOI:** 10.3389/fpls.2023.1320660

**Published:** 2024-01-05

**Authors:** Yu-Ru Chen, Thomas Lübberstedt, Ursula K Frei

**Affiliations:** ^1^ Department of Agronomy, Iowa State University, Ames, IA, United States; ^2^ Crop Science Division, Taiwan Agricultural Research Institute, Ministry of Agriculture, Taichung, Taiwan

**Keywords:** doubled haploids, haploid inducers, *in vivo* induction, line development, transgressive segregation

## Abstract

Haploid inducers are key components of doubled haploid (DH) technology in maize. Robust agronomic performance and better haploid induction ability of inducers are persistently sought through genetic improvement. We herein developed *C1-I* inducers enabling large-scale *in vivo* haploid induction of inducers and discovered superior inducers from the DH progenies. The haploid induction rate (HIR) of *C1-I* inducers ranged between 5.8% and 12.0%. Overall, the success rate of DH production was 13% on average across the 23 different inducer crosses. The anthesis–silking interval and days to flowering of inducer F_1_s are significantly correlated with the success rate of DH production (r = −0.48 and 0.47, respectively). Transgressive segregants in DH inducers (DHIs) were found for the traits (days to flowering, HIR, plant height, and total primary branch length). Moreover, the best HIR in DHIs exceeded 23%. Parental genome contributions to DHI progenies ranged between 0.40 and 0.55, respectively, in 25 and 75 percentage quantiles, and the mean and median were 0.48. The allele frequency of the four traits from inducer parents to DHI progenies did not correspond with the phenotypic difference between superior and inferior individuals in the DH populations by genome-wide Fst analysis. This study demonstrated that the recombinant DHIs can be accessed on a large scale and used as materials to facilitate the genetic improvement of maternal haploid inducers by *in vivo* DH technology.

## 1 Introduction

Doubled haploid (DH) technology has become an established tool in modern plant breeding to rapidly produce homozygous lines. This is accomplished by producing haploid plants with only one set of chromosomes, followed by the doubling of their haploid genomes. DH technology offers numerous benefits, such as a more efficient selection process compared to selection among segregating families, and reducing the time and costs involved in inbred line development by repeated self-pollination. The homozygous nature of DH lines allows for uniform and consistent plant populations, enabling breeders to accurately evaluate the performance of different genetic materials in breeding programs.

Successful implementation of DH technology in maize breeding programs depends on the ability to produce haploids. This is achieved by using pollen from haploid inducers to pollinate the source germplasm from which DH lines will be developed (Chang and Coe, 2009; Chaikam et al., 2019b). The proportion of seeds with haploid embryos detected among the total seeds harvested on the source germplasm is referred to as the haploid induction rate (HIR) (Prigge et al., 2012a). Over the past 15–20 years, more advanced inducers with a high HIR (8%–10%) have been developed based on the original Stock 6 genotype and enabled the production of maize DH lines at a large scale (Geiger and Gordillo, 2009). Improved modern inducers include RWS, RWK-76, UH400, and UH600 (University of Hohenheim, Germany), the PHI series (Procera, Romania), CAU5 (China Agricultural University, China), the BHI series (Iowa State University, USA), and the TAIL series (Tropical Adapted Inducer Lines, CIMMYT) (Geiger and Gordillo, 2009; Rotarenco et al., 2010; Prigge et al., 2012b; Xu et al., 2013; Chaikam et al., 2018; Trentin et al., 2020; Trentin et al., 2023).

Genetic studies of HIRs revealed that it is a quantitative trait controlled by a few major quantitative trait loci (QTLs) along with minor QTLs (Barret et al., 2008; Prigge et al., 2012b). Two major QTLs, *qhir1* and *qhir8*, were shown to be responsible for HIRs in QTL mapping studies (Prigge et al., 2012b; Xu et al., 2013). Later, the gene underlying *qhir1* was cloned by three independent research groups simultaneously and called *MATRILINEAL*, *ZmPHOSPHOLIPASE-A1*, or *NOT LIKE DAD* (*MTL/ZmPLA1/NLD*) (Gilles et al., 2017; Kelliher et al., 2017; Liu et al., 2017). *MTL/ZmPLA1/NLD is* a pollen-specific gene encoding for a putative phospholipase. A 4-bp insertion mutation in *MTL* (we will use only this name for simplicity) causes a frameshift and a truncated protein. The presence of a single nucleotide change in the gene *ZmDMP* in *qhir8* has been shown to increase HIRs two- to threefold when *MTL* is present. However, *ZmDMP* has a very low HIR (~0.15%) on its own (Zhong et al., 2019). The synergistic effect of *mtl* and *zmdmp* mutations suggests that interactions of gene functions after pollination contribute to a high HIR in modern inducers (Jacquier et al., 2020). Furthermore, a loss-of-function mutation of *Zea mays PHOSPHOLIPASE D3* (*ZmPLD3*) induced by the CRISPR-Cas9 machinery showed synergistic effects, increasing HIRs threefold (from 1.19% to 4.13%) in the presence of *mtl* (Li et al., 2021). Pollen reactive oxygen species (ROS) bursts lead to sperm DNA fragmentation with an impact on haploid induction, as observed in the *mtl* genotype. In accordance with this finding, mutants of *ZmPOD65* discovered as a peroxidase gene in the ROS class induced by CRISPR-Cas9 machinery contributed to the aborted kernel, and the HIRs were between 0.9% and 7.7% (Jiang et al., 2022). Additional genome regions and underlying candidate genes have been identified as putatively controlling HIRs in a genome-wide association study (e.g., Trentin et al., 2023). A novel gene in the *Arabidopsis* model plant, *AtKPl* (*KOKOPELLI*), which is only expressed in male gametophytes, has been discovered to play a role in single fertilization events to trigger maternal haploid induction (Jacquier et al., 2023). Although the biological mechanism of haploid induction in maize remains ambiguous, effective methods for accumulating favorable alleles of other genes affecting HIRs in fixed *mtl* genetic backgrounds are required for further genetic improvement of inducers and biological studies of haploid induction.

The most cost-effective way to sort haploids from diploids is visual selection at the seed level, followed by the seedling stage (Vanous et al., 2017; Dermail et al., 2023). Anthocyanin biosynthesis structural genes such as *A1*, *A2*, *C2*, *Bz1*, *Bz2*, and *Pr1* are regulated by the *MYB C1* (colored aleurone1)/*Pl1* (purple plant1) and *bHLH R1* (coloured1)/*B1* (plant colour1) gene families. Since each member of these families has a tissue- or development-specific expression, the anthocyanin pigmentation pattern of a maize plant and kernel depends on the allelic constitution at the *C1/Pl1* and *R1/B1* loci. For example, *C1* gene is responsible for anthocyanin development in the aleurone layer of the maize kernel, while *Pl1* gene is associated with sun-independent anthocyanin pigmentation in plant tissues and the pericarp of the maize kernel. Likewise, *R1* gene activates anthocyanin pigmentation of plant tissues and the aleurone layer in maize kernel, but *B1* sun-dependently regulates plant color and is expressed in the pericarp of the maize kernel (Walker et al., 1995; Petroni et al., 2014). The most common haploid kernel identification marker is the purple crown coloration of endosperm and purple coleoptiles of embryos encoded by the dominant mutant allele *R1-nj* of the red color gene *R1* (Nanda and Chase, 1966). Current inducers contain *R1-nj* gene for identifying donor haploids at the seed stage. In the aleurone, in which cells have one copy of the *R1-nj* allele, anthocyanin pigments are expressed and allow the identification of contaminations or self-pollinations without coloration. Within the fraction of kernels with a purple kernel crown (usually the majority in controlled induction crosses), haploids are identified by the absence of red coloration of the embryo (Prigge and Melchinger, 2012). The intensity and extent of pigmentation can vary depending on the donor’s and inducer’s genetic backgrounds. For example, in the presence of *B1* and *Pl1* color genes, *R1-nj* positively affects the pigmentation of the coleoptile, root color, and plant color. However, if *C1-I* gene is present in either donor or inducer, the pigmentation of the coleoptile and aleurone layer of maize kernels disappears because *C1-I* gene inhibits the *R1-nj* function epistatically in the biosynthesis of anthocyanin in maize kernels. While this is an impediment in regular induction crosses, inhibition of *R1-nj* by *C1-I* can be exploited to enable the identification of haploids in genetic backgrounds carrying *R1-nj* gene, such as inducers. If conventional inducers are crossed with inducers carrying *C1-I* gene, the resulting F_1_ (*R1-nj* × *C1-I* inducer cross) is expected to have no anthocyanin pigmentation of the embryo unless the embryo has the maternal haploid genotype.

The objectives of this study were to i) verify that indeed maternal inducer haploids can be produced by *in vivo* haploid induction using a *C1-I* inducer, ii) demonstrate that DH inducer (DHI) line development is feasible, iii) determine the relationship between traits of inducer F_1_s and the success rate of DHI production, iv) understand the relationship between HIRs and agronomic traits in DHI progeny populations, and v) evaluate the ability to accumulate favorable alleles from inducer parents in superior inducers through DH technology.

## 2 Materials and methods

### 2.1 DH technology applied to DH inducer line development

**Figure 1:**
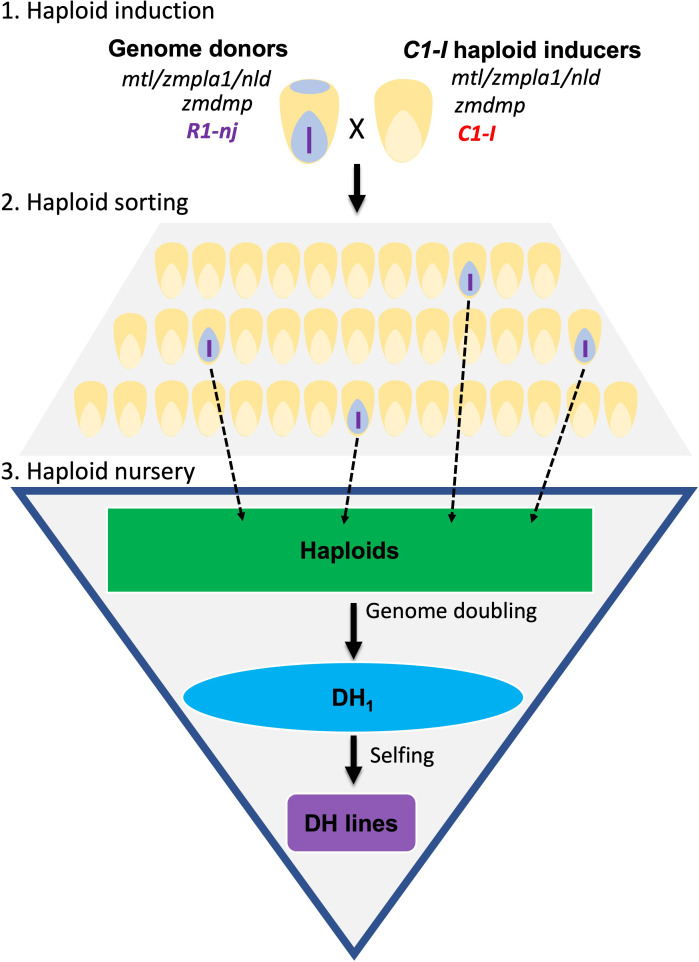
Doubled haploid (DH) technology for DH inducer line development. The diagram illustrates three main steps to obtain DH inducer (DHI) lines. Step 1: C1-I inducers were used as males to cross with regular inducer F_1_s for the haploid induction. Step 2: sorting the maternal haploids with the purple embryo expressed by R1-nj. Step 3: treating the inducer haploids with colchicine for genome doubling in greenhouse, then transplanting haploid seedlings to a field nursery, and finally obtaining the DHI lines after self-pollination.

There are three stages in the DHI production pipeline (**Figure 1**). First, heterozygous inducer F_1_s used as donor parents were induced by a *C1-I* inducer for haploid induction. Second, putative haploid kernels expressing a purple color in the embryo need to be visually sorted. Third, haploid kernels are sown in the greenhouse, and haploid seedlings are injected with colchicine solution for genome doubling at the three-leaf stage (Vanous et al., 2017). Colchicine-treated haploids are subsequently transplanted in the field and self-pollinated to obtain DHI lines. The success rate reflects the percentage of transplanted haploids with seed sets leading to DHI lines.

### 2.2 *C1-I* inducer development

The public line Mo47 served as the donor of the *C1-I* allele. Crosses with three different haploid inducer lines (RWS, BHI306, and BH201) were subsequently performed to combine haploid induction ability with the presence of the *C1-I* allele. Upon each cross with an inducer line, a generation of self-pollination followed to select ears fixed for the *C1-I* allele. The resulting families were continued by ear-to-row and selected for their agronomic performance and haploid induction ability in a genetic background fixed for the *R1-nj* allele. In this study, five F4 generation inducer genotypes were selected for their HIR performance and used for DHI line development.

### 2.3 Application of *C1-I* inducers for *R1-nj* inducer development

Eight inducers were used to develop an elite-by-elite DHI population (nos. 1–8 in **Table 1**). These inducers were selected from the crosses of RWS/RWK76 and exPVP/public maize inbred lines, which were fixed for *mtl* and *zmdmp* genes. A total of 28 inducer F_1_s were created from the eight inducers by a half-diallel mating design in the summer of 2019 and evaluated for haploid induction ability and agronomic performance in the summer of 2020. B73- and PHG83-derived inducers were crossed with MHI (carrying *mtl*, but not *zmdmp*) to create elite-by-traditional (HIR< 5%) inducer combinations. The inducer F_1_s were crossed with *C1-I* inducers to obtain inducer haploids. Inducer haploids were subjected to the regular protocol of DH technology in maize (Vanous et al., 2017; Aboobucker et al., 2022) to obtain DHI lines. In addition, the HIRs of five *C1-I* inducers were evaluated by crossing them with nine inducer F_1_s and BHI306 and B73_*R1-nj* inbred (Maize Genetic Stock center: X17D), which were used as checks in the summer of 2020.

**Table 1:** Agronomic and HIR performance of haploid inducer parents.

No.	Inducer F_1_ parents	Phenotypic markers	Traits			
			DTF	HIR	PHT	PBL
**1**	BHI306	*R1-nj*, red root	60.4 (7)^*^	14.6 (3)	138.2 (6)	130.7 (6)
**2**	Mo17-derived	*R1-nj*, red root	64.0 (5)	16.1 (1)	168.0 (2)	117.8 (8)
**3**	PHG83-derived	*R1-nj*	64.2 (3)	12.6 (7)	154.4 (5)	290.6 (1)
**4**	FR19-derived	*R1-nj*	61.2 (6)	13.9 (4)	176.3 (1)	178.6 (2)
**5**	A637-derived	*R1-nj*, red root	59.7 (9)	13.4 (5)	134.8 (8)	151.0 (3)
**6**	B84-derived	*R1-nj*, red root	65.0 (2)	12.3 (8)	159.3 (4)	143.2 (5)
**7**	LH82-derived	*R1-nj*	60.0 (8)	12.8 (6)	135.5 (7)	150.0 (4)
**8**	B73-derived	*R1-nj*	66.4 (1)	15.1 (2)	168.0 (3)	88.1 (9)
**9**	MHI	*R1-nj*	64.1 (4)	3.5 (9)	131.3 (9)	130.1 (7)

DTF, days to flowering; HIR, haploid induction rate (%); PHT, plant height (cm); PBL, total primary branch length (cm).

*Numbers in the parentheses are the performance of the inducer F_1_ parents arranged by rank.

### 2.4 Plant materials and experimental design

Phenotypes of inducer parents and DHI lines were determined in the summers of 2021 and 2022. However, phenotypes of the *C1-I* inducers and inducer F_1_s were only measured in the summer of 2020. A commercial F_1_ hybrid, Viking 60-01N (released by Albert Lee Seed Company, MN), was used as the donor for testing HIRs of inducers. Plant materials were planted in 3.8-m plots by randomized complete block design in two planting blocks at the Iowa State University Agricultural Engineering and Agronomy Farm in Boone, IA. A total of 537 genotypes were planted in the field for phenotypic measurement in 2021 and 2022. The entries were unbalanced but 42 DHI genotypes, and nine parent founders were common across two years. All trials were sown in loamy soil under rainfed conditions, adopting standard agronomic practices for research at Iowa State University.

### 2.5 Phenotypic measurements

Four phenotypic traits were evaluated. Plant height was used as a measure of the agronomic performance of the inducer line and traits influencing immediately the performance of a haploid inducer line: days to flowering, tassel branch length, and HIRs during haploid induction in the field. Days to flowering (DTF) was measured as 50% of plants per plot shedding pollen. Days to silking (DTS) was equivalent to 50% of plants in plots that were silking. The anthesis–silking interval (ASI) was the difference between DF and DS. Plant height was measured as the distance (cm) from the ground to the flag leaf’s base after pollen shedding. The total primary branch length (PBL) was used to represent the tassel size of inducers, which was measured as the cumulative length (cm) of all primary tassel branches. HIRs were calculated using the number of haploid kernels divided by the total number of seeds from at least five donor cobs induced by the inducer genotypes, which were harvested after the R6 stage. Suspicious haploid kernels were cut open using a scalpel to reveal the embryo color underneath the pericarp. Entries with fewer than 800 kernels for HIR evaluation were excluded to ensure high HIR data quality (Chaikam et al., 2018). After phenotypic measurements and data collection, the number of DHIs obtaining phenotypes in this study for DTF, HIRs, PHT, and PBL were 334, 331, 338, and 537, respectively.

### 2.6 SNP genotyping

Seeds of DHI lines were sown in the agronomy greenhouse at Iowa State University. Leaf tissue from three plants per entry was harvested, the tissue was lyophilized for 24 hours, and leaf samples were shipped to CIMMYT in El Batán, Texcoco, Mexico, for DNA extraction and single-nucleotide polymorphism (SNP) genotyping using DArTseq technology (Jaccoud et al., 2001). A total of 88,421 unimputed SNPs per line were successfully called and reported. Allele sequences were blasted against the B73 reference genome (B73 RefGen_v4) to obtain unique SNP positions to generate the HapMap files. SNP HapMap files were transformed into VCF files and numeric genotype files in TASSEL Version 5.0 (Bradbury et al., 2007). The missing SNP genotype imputation was conducted using Beagle 5.4 software (Browning et al., 2018). Also, minor allele frequencies of SNPs with less than 5% were filtered out. In total, 6636 SNPs across the genome remained and were used for calculating parental genome contribution and fixation index (Fst) in this study. The SNP genotypes of lines were coded as (−1 and 1), where 1 represents homozygosity for the major allele at a given bi-allelic locus and −1 indicates homozygosity for the minor allele.

### 2.7 Statistical analysis

The least-square mean of the trait performances (responses) in the field trial for genotypes was estimated by the following equation:


$${\text{y}}_{ijk}=\text{μ}+{\text{genotype}}_{i}+\text{block}{\left(\text{year}\right)}_{\text{j}(\text{k})}+{\text{year}}_{\text{k}}+{\text{ε}}_{i\text{jk}},$$

where *y*_*ij*k_ is the response for genotype i in block j nested in year k, μ is the overall mean, genotype_*i*_ is the fixed effect of genotype, block(year)_*j*k_ is the fixed effect for block, year_k_ is the fixed effect for a year, and ε_*ij*k_ is the residual, which is independent and identically normal distributed N(0, σ_ε_^2^). The least-square mean of responses of genotypes, which are represented as best linear unbiased estimator (BLUE) values, were estimated using the emmeans package (Searle et al., 1980). To obtain broad-sense heritability (H^2^) on a plot basis, σ_g_^2^/(σ_g_^2^+σ_ε_^2^/r) was calculated, where r is the harmonic mean of replicates of DHIs (Holland et al., 2010). Genotype effects were treated as random to estimate σ_g_^2^ using the lmer package (Bates et al., 2015).

P_1_ and P_2_ are considered the parental lines of the DHI progenies, and (x_1_, x_2_, x_DHI_) represents their SNP genotype matrix. The (m × 1)-dimensional column vector *β* of effects follows parental identity by descent (IBD) contribution to progeny considering only polymorphic loci between P_1_ and P_2_, as follows:


$$\beta =\frac{x_{1}-x_{2}}{{\left(x_{1}-x_{2}\right)}^{'}\left(x_{1}-x_{2}\right)}$$

The mean of parental genome contributions in the DHI progeny was computed as


$$PGC_{DHI}=x_{DHI}^{'}\beta +0.5.$$

Fst was used to measure the level of genetic differentiation of genome-wide SNPs between two populations in this study, calculated using the gwscaR package (Flanagan and Jones, 2017). In order to understand genetic differentiation after applying DH technology in inducer line development, inducer individuals were classified into groups depending on the pedigree or phenotype. Based on trait performance, three DHI parents were chosen as reference lines. BHI306 was a relatively early flowering and short plant. A PHG83-derived inducer showed a relatively low HIR but had large tassels. A B73-derived inducer was late flowering, with high HIRs and a tall plant but small tassels (**Table 1**). In terms of the phenotype and genetic source difference of the reference lines, the DHI progenies from their F_1_ combinations were divided into three nested DHI populations, similar to the nested association mapping (NAM) mating structure. Genome-wide SNP Fst analysis was conducted between the group of DHI parents and their DHI progeny (i.e., based on pedigree) and the group of between phenotypically superior and inferior DHIs for every single trait of interest. The top and bottom two individuals were selected within each family (i.e., based on phenotype).


$$Fst=\frac{\bar{p}\left(1-\bar{p}\right)-\sum c_{i}p_{i}\left(1-p_{i}\right)}{\bar{p}\left(1-\bar{p}\right)}$$

In the equation above, $\bar{p}\left(1-\bar{p}\right)$ is the expected heterozygosity of a bi-allelic locus when the two populations are considered as one large meta-population. Moreover, $\sum c_{i}p_{i}\left(1-p_{i}\right)$ is the average expected heterozygosity for each group within a pedigree-based or phenotype-based population. Fst values range from 0 (no genetic differentiation) to 1 (complete genetic differentiation).

Considering the genetic architecture of the traits of interest were not controlled by an infinitesimal (polygenic) model in this study, the quantile 99% (Q99) was chosen as the threshold to detect outlier Fst values of SNPs. SNPs that have extreme Fst values (Q99 SNPs) in pedigree-based population comparison are the most likely candidates to be affected by any factors and natural selections during the DH technology process. However, the Q99 SNPs in phenotype-based comparison are probably the genome region associated with the phenotypic difference. The sensitivity statistic was used to understand the ratio of the same SNPs detected in the Fst analysis based on both pedigree and phenotype. t-tests were used to test if the sensitivity was greater than zero and the mean differences between groups were equal to zero (null hypothesis) using the rstatix package.

## 3 Results

### 3.1 Inducer haploid induction and sorting

**Figure 2:**
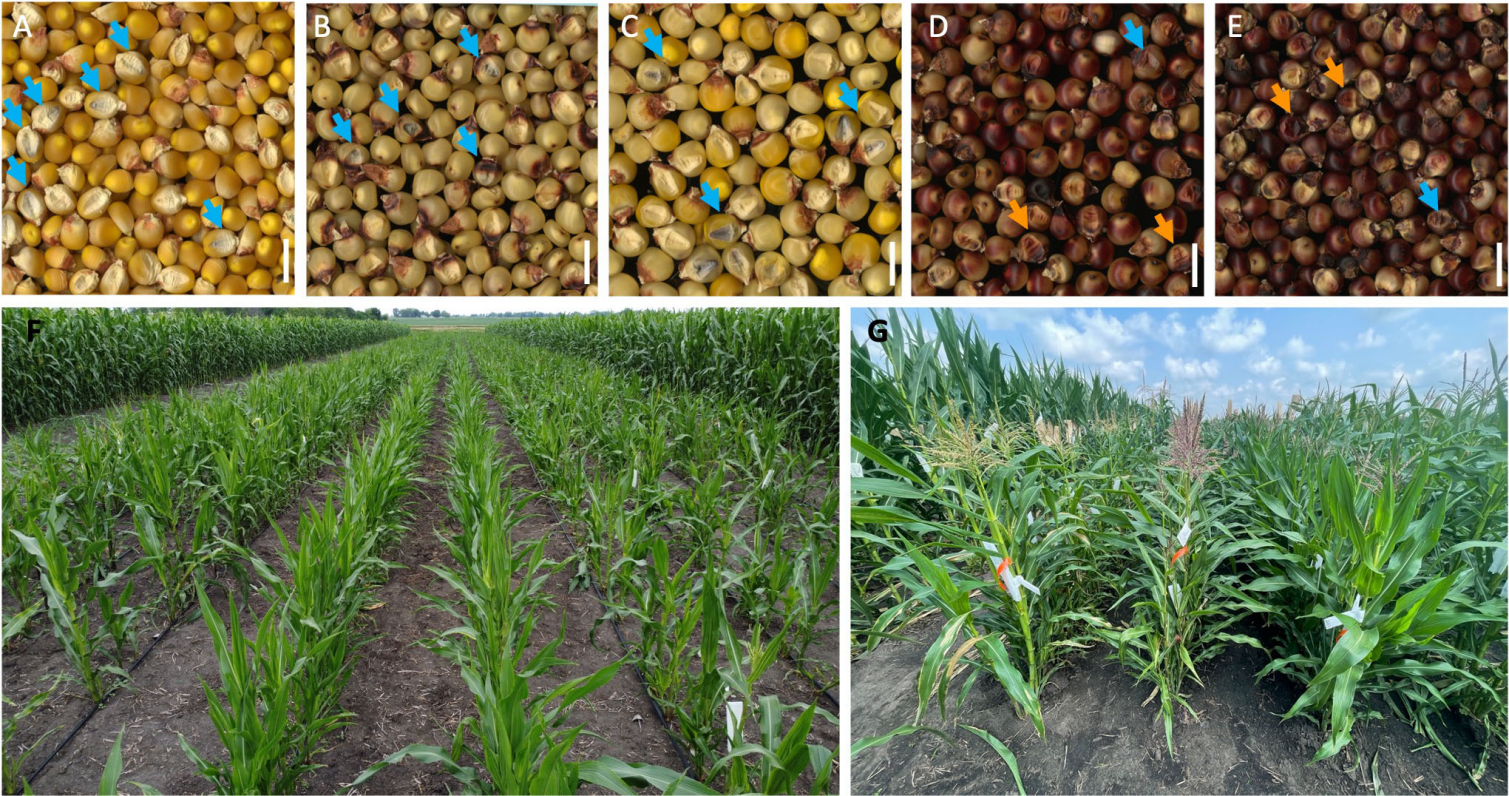
Pigmentation of kernels induced by C1-I inducers and doubled haploid inducer (DHI) agronomic performance. **(A)** B73_R1-nj inbred donor (check1). **(B)** B73-derived inducer/BHI306 inducer F_1_ donor. **(C)** B73-derived inducer/B84-derived inducer F_1_ donor. **(D)** Mo17-derived inducer/B84-derived inducer F_1_ donor. **(E)** BHI306 inbred donor (check2). Light blue arrows point to haploids with clear anthocyanin pigmentation, while orange arrows point to suspicious haploids with vague purple-red pigmentation. The white bar represents 1 cm. **(F)** Large-scale inducer haploid plant nursery in the field for DHI production. **(G)** Phenotypic performance of DHI lines in trials.

The LS means of HIRs for the five *C1-I* inducers ranged from 5.8% to 12.0%, and HIRs differed significantly between *C1-I* inducers (**Table 2**). The inducer parents used in this study, which have the red root (*Pl1*) phenotypic marker for haploid sorting, are BHI306, Mo17-derived, A637-derived, and B84-derived inducers (**Table 1**). The pigmentation of kernels of inducer F_1_s after haploid induction by *C1-I* inducers showed that the pigmentation expression depended on the inducer F_1_ genotypes. Color pigmentation patterns are illustrated by the representative combinations (**Figures 2A–E**). The kernels of B73-derived/BHI306 and B73-derived/B84-derived inducer F_1_s after haploid induction by *C1-I* inducer had similar pigmentation patterns to the check B73_*R1-nj* inbred, which did not show purple-red endosperm pigmentations of the putative diploid. In contrast, the kernels of Mo17-derived/B84-derived inducer F_1_ had purple-red pigmentations on all of the kernels after induction, which were like induction outcomes of the check BHI306 inducer inbred. The putative haploids could still be sorted based on anthocyanin pigments in the scutellum. However, the pigmentation of the region around the embryo of the kernel increased the proportion of the suspicious haploids in haploid sorting.

**Table 2:** The LS mean of HIR by five *C1-I* haploid inducers.

*C1-I* inducer genotype	LS mean	95% confidence interval	
		Lower	Upper
A	9.1^abc^	5	13.1
B	12.0^c^	8.6	15.3
C	5.8^a^	2.5	9.2
D	10.9^bc^	7.5	14.2
E	6.1^ab^	2	10.2

HIR of C1-I inducer was shown by percentage (%). LS mean values not sharing any letter in the LS mean column are significantly different by Tukey’s test at the 5% level of significance.

HIR, haploid induction rate.

### 3.2 Inducer haploid nursery

**Figure 3:**
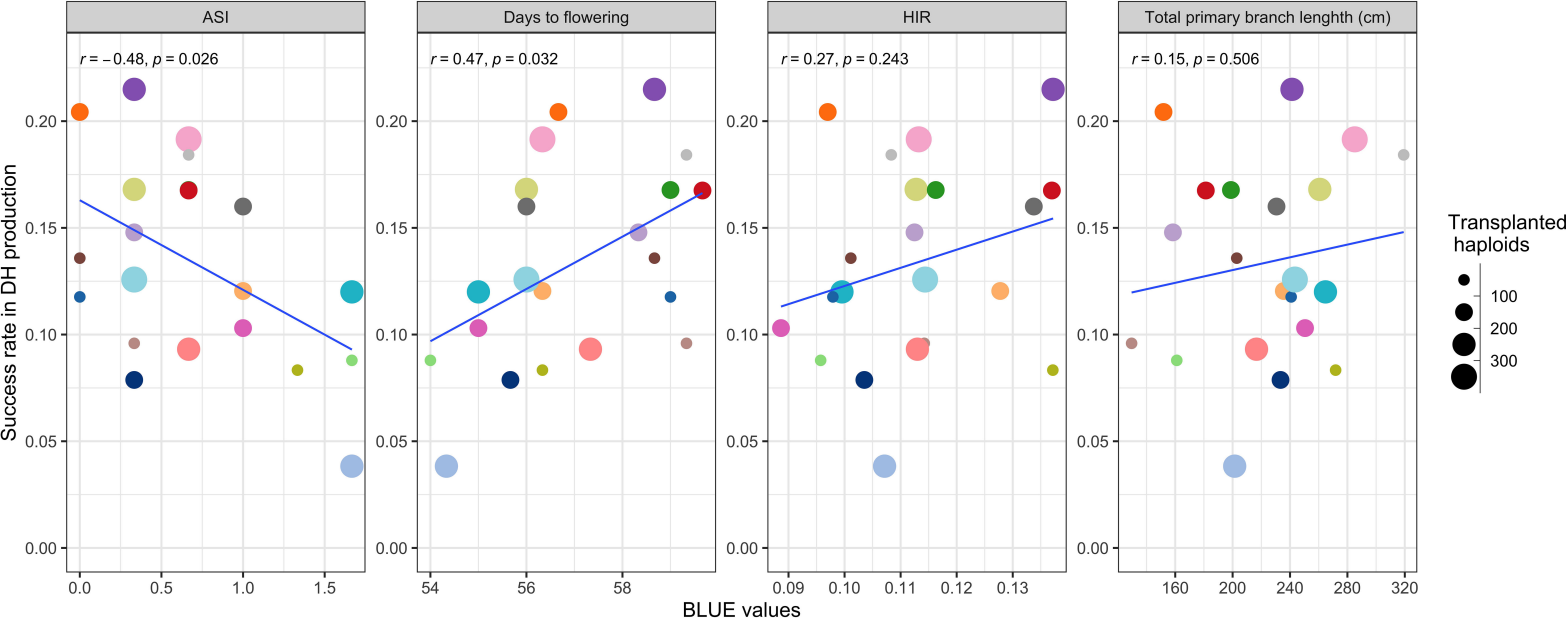
The relationship of the success rate of doubled haploid (DH) production between traits of different inducer F_1_ genome donors.

Applying DH technology for inducers within two seasons allowed us to obtain 100% homozygous DHI populations with uniform agronomic performance (**Figure 2G**). The HIRs of *C1-I* inducers (genotypes A, B, and D) exceeded 8%, which enables large-scale DHI production (**Figure 2F**). Of inducer haploid seedlings treated with colchicine solution for genome doubling, 22% were shedding pollen, with a 13% success rate on average based on initially transplanted inducer haploids to obtain DHI lines in this study. The ASI of inducer F_1_ genome donors was significantly negatively correlated with the success rate (r = −0.48, p-value = 0.03), and the DTF of inducer F_1_ genome donors was significantly positively correlated with the success rate (r = 0.47, p-value = 0.03). There was no evidence that HIRs and PBL of inducer F_1_s were correlated with success rate in DHI production (**Figure 3**).

### 3.3 Phenotypic performance of DHI progenies

**Figure 4:**
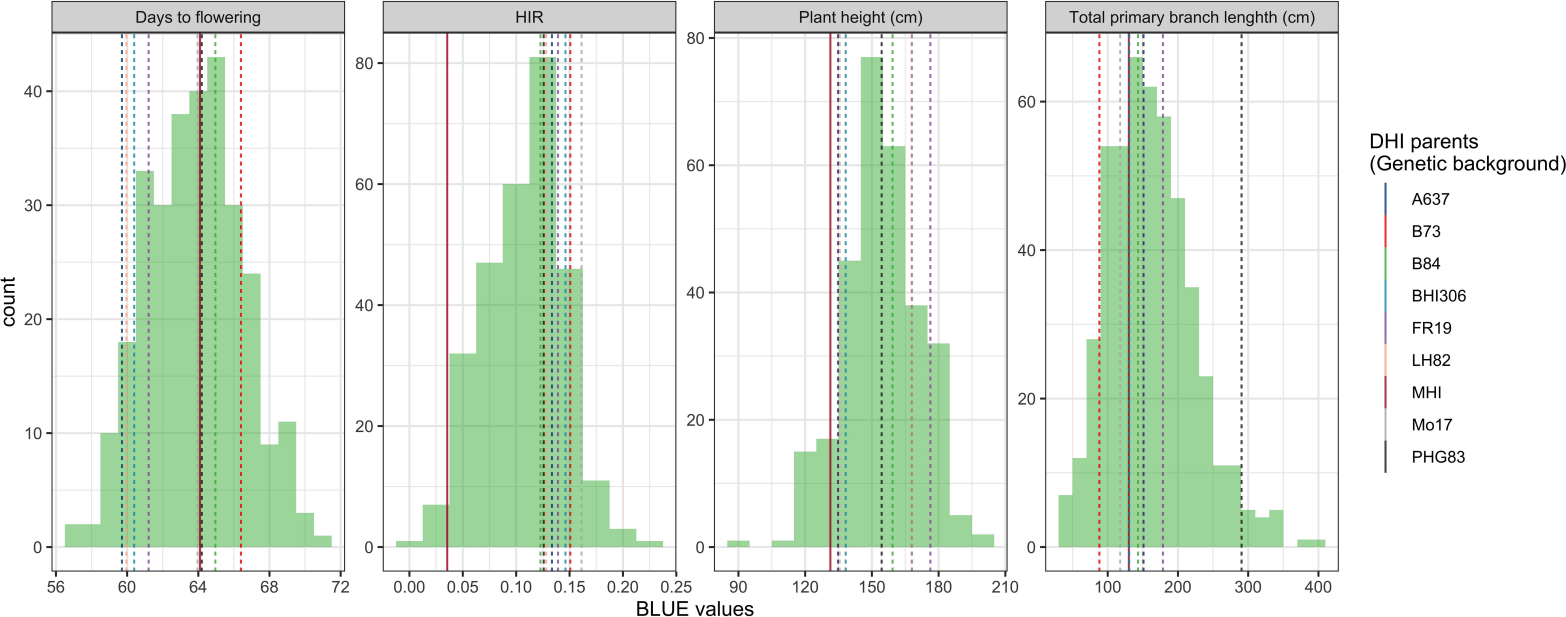
Phenotypic distribution of four traits in the doubled haploid inducer (DHI) sample population. The dashed lines represent the performances of the eight elite inducer parents, which had more than 10% haploid induction rate (HIR). The solid line represents the performances of traditional inducer (MHI).

The major QTL alleles of haploid induction ability of the eight DHI parents derived from different exPVP/public genetic background lines were contributed by the RWS inducer, while the HIRs of DHI parents all exceeded 12%, and the other three agronomic traits varied among DHI parents (**Table 1**). The estimated mean of the four trait performances between the DHI parents and the sample of DHI progenies was not significantly different (**Table 3**). The ranges of maximum and minimum performances of the four traits in DHI progenies were wider than those of DHI parents (**Table 3**), indicating transgressive segregation (**Figure 4**). Moreover, the mean differences between inferior and superior groups of DHIs were significant (p< 0.05) for the four traits (**Table 3**).

**Table 3:** Trait performance of inducer parents and DHI lines.

Statistics	Traits			
	DTF	HIR	PHT	PBL
H^2^	0.75	0.66	0.72	0.59
Max (parents)	66.4	16.1	176.29	290.57
Max (DHIs)	70.52	23.4	202	407.12
Min (parents)	59.71	3.5	131.33	88.14
Min (DHIs)	56.9	0.4	93.96	32.52
Mean (parents)	62.76	12.7	151.76	153.33
Mean (DHIs)	63.56	10.75	154.43	162.37
Mean difference	0.80	−1.95	2.67	9.04
Mean (inferior group)	60.65	7.46	134	85.05
Mean (superior group)	66.22	15.61	175.41	252.22
Mean difference	−5.57*	−8.15**	−41.41***	−167.17*
Sensitivity of detected SNP in Fst analysis	0.035	0.011	0.010	0.005

DTF, days to flowering; HIR, haploid induction rate (%); PHT, plant height (cm); PBL, total primary branch length (cm); SNP, single-nucleotide polymorphism.

*<0.05; **<0.01; ***<0.001 (the alpha significant level).

The sensitivity statistic was the mean proportion of the same SNPs detected in the Fst analysis based on both pedigree and phenotype from the SNPs detected based on phenotype in the three samples of nested association mapping (NAM) populations.

### 3.4 Parental genome contribution and genetic differentiation in DHI populations

**Figure 5:**
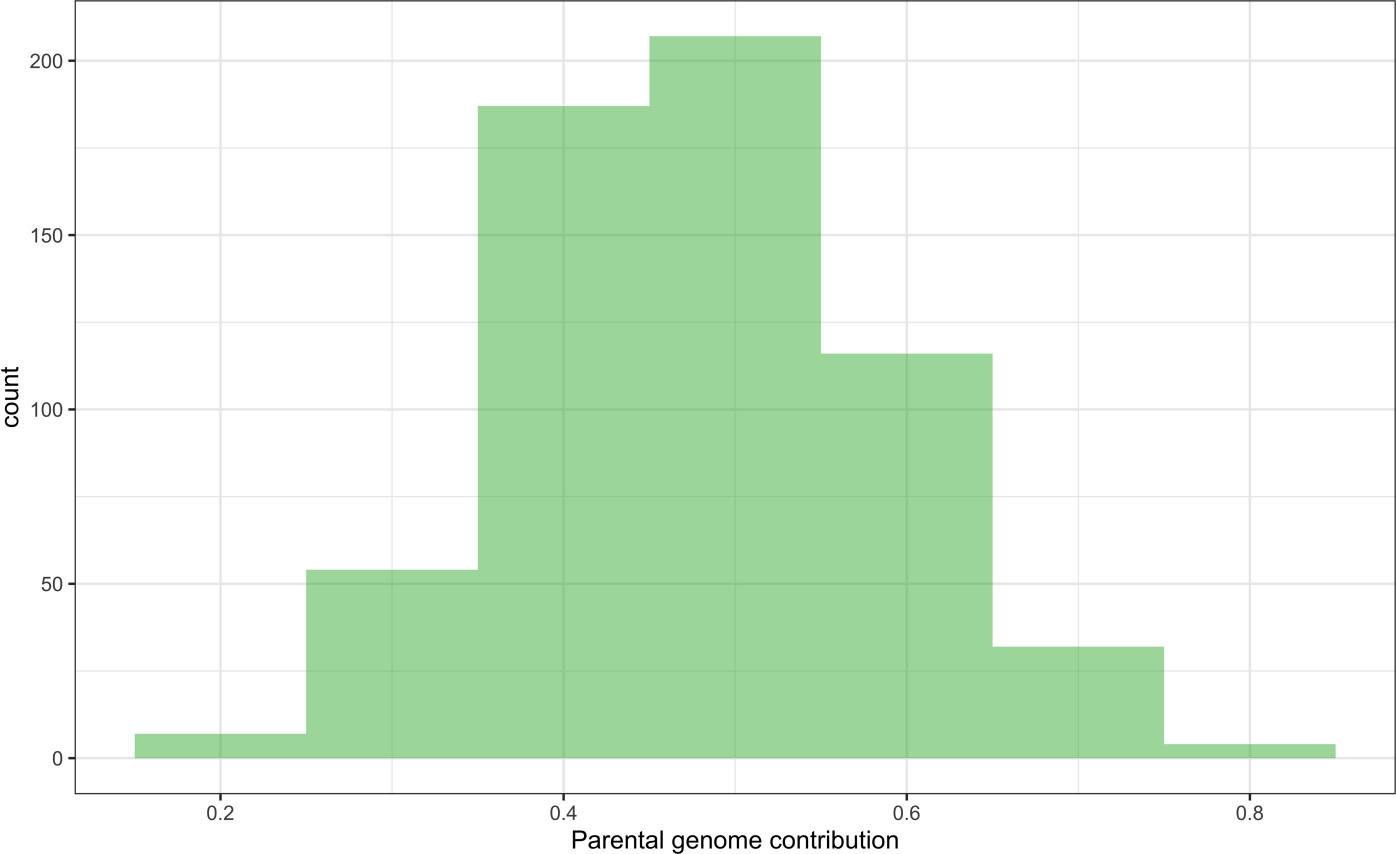
The distribution of parental genome contribution to doubled haploid inducers (DHIs) derived from the 23 biparental crosses.

**Figure 6:**
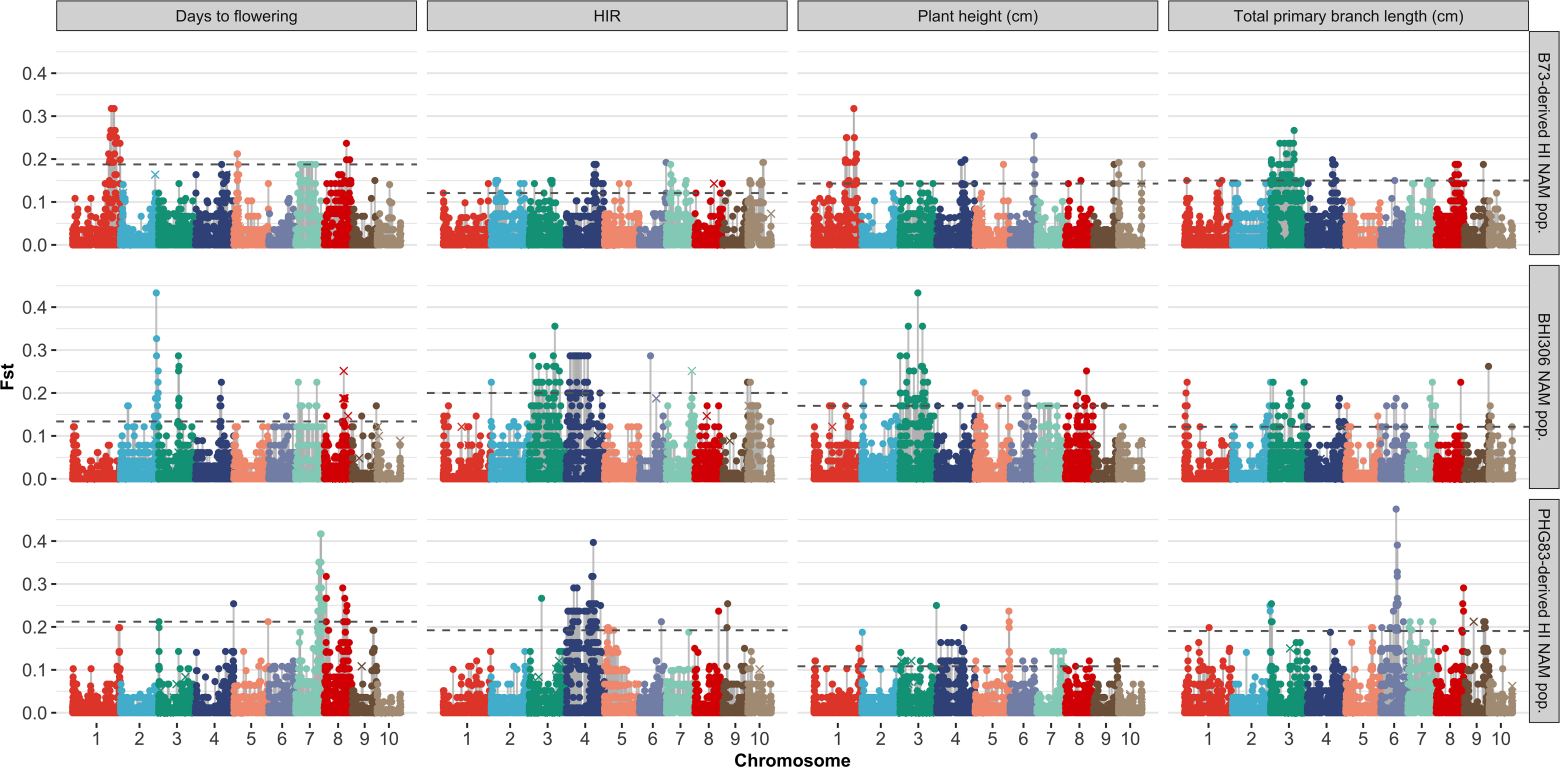
Genome-wide SNP Fst analysis of the traits of superior versus inferior doubled haploid inducers (DHIs) from three samples of inducer-derived nested association mapping (NAM) populations. The horizontal dash line was the Q99 threshold used to detect the outlier Fst SNPs. The × symbol represents Q99 outlier Fst SNPs detected from the groups of parents and their respective derived DHI progenies. The three lines chosen as the reference lines in the NAM samples were BHI306, which had relatively early flowering and short plant; PHG83-derived inducer, which had relatively low haploid induction rate (HIR) but big tassels; and B73-derived inducer, which had late flowering, relatively high HIR, and tall plant but small tassels.

The majority of DHIs obtained approximately equal genome contributions from both parents (**Figure 5**). The first quantile, mean/median, and third quantile of parental genome contribution were 0.40, 0.48, and 0.55, respectively. Most of the SNP outliers, which exceeded the 99% quantile (Q99 SNPs), were dependent on the population (**Figure 6**). This means that extraordinary individuals from the three NAM populations had different SNP genotypes associated with phenotypic differences. Comparing Q99 SNPs detected in both pedigree and phenotype groups, the mean sensitivity of Q99 SNPs was below 0.05 for the four traits (**Table 3**). There is, therefore, no evidence that DHI production was affected by a change in allele frequencies when considering the four traits between superior and inferior DHI lines (**Figure 6**).

## 4 Discussion

### 4.1 Recombination for more genetic variation

Chaikam et al. (2018) observed that crosses of haploid inducer lines with agronomical superior and environmentally well-adapted non-inducer lines not only improved the agronomic performance of the existing inducer but also had a positive effect on HIRs. The DHI parents in this study were derived from crosses between the haploid inducer line RWS and several ex-PVP/public non-inducer inbreds. Transgressive segregants were detected for all four quantitative traits including HIRs, when samples of more than 50 inducer haploid plants were transplanted per cross (**Figures 3**, **4**). Moreover, the best DHI progenies from the parental crosses (PHG83-derived/BHI306 and PHG83-derived/Mo17-derived) with an HIR > 13.9% on average outperformed their parental lines with induction rates exceeding 20% (**Figure 4**). The majority of parental genome contributions to DHIs ranged from 0.40 to 0.55. In conclusion, elite × elite inducer crosses appear promising to derive even better performing DHIs.

The DHI lines in this study were obtained from maternal haploids, which had the *mtl* and *zmdmp* alleles fixed. Thus, other genes must be responsible for increasing haploid induction ability in our DHIs. Those could be the more recently identified HIR genes (Li et al., 2021; Jiang et al., 2022; Jacquier et al., 2023). However, Trentin et al. (2023) and others have shown that there is additional genetic variation available for HIRs, in addition to the four identified genes to date. Thus, it appears promising to combine DHI technology with genomic selection, while accumulating known HIR genes, to develop superior maternal haploid inducers in the future, with HIRs >20% (observed in this study) or even >25%.

DHIs in this study were obtained from the induction of inducer F_1_s (**Figure 1**). While only a single recombination happens in the F_1_ generation, it still resulted in significant phenotypic mean differences between superior and inferior DH line families (**Table 3**). This suggests that obtaining DHIs from inducer F_1_ generation is sufficient to create substantial phenotypic variation for selection. This is consistent with Sleper and Bernardo (2016), who reported that superior DHs can be obtained from inducing F_1_ rather than F_2_ generations.

### 4.2 Benefits for line development and selection

There is a continuous need for further improved haploid inducers to enhance the efficiency of DH technology in maize overall and to adapt haploid inducers to different environments (Trentin et al., 2020; Dermail et al., 2021). Applying DH technology not only shortens the time needed for inducer line development but also allows for more efficient genotypic selection for qualitative traits using haploid plants or DHIs, rather than segregating inducer families (Lübberstedt and Frei, 2012). For example, color appearance traits controlled by *B1* and *Pl1* anthocyanin regulatory genes, common rust, and leaf blight major resistance genes (Prigge et al., 2012a) are straightforward to be observed and selected for in the haploid nursery (**Figure 2**).

The overall success rate for DH production ranges from 10% to 22% for Dent Corn using artificial genome doubling by colchicine (Melchinger et al., 2016). The success rate of DH production in the haploid inducer nursery ranged from 3.8% to 21.5%, with 13.3% on average (**Figure 3**). Thus, the efficiency of colchicine application for artificial genome doubling in Dent Corn and inducers was similar. A negative correlation between the success rate of DH production and ASI of inducer F_1_s was observed. ASI of lines is an important indicator to assess the ability of flowering and nicking in water-deficit environments (Ribaut et al., 1996; Sah et al., 2020). Selecting closer ASIs of inducer F_1_s has the potential to increase the success rate of DH production and obtain DHI progenies with better drought tolerance.

The DHI line development in this study depends on the selection of *R1-nj* expression on the kernels of female parents (**Figure 1**). When using non-inducer inbreds as genetic sources to improve the inducer breeding population, *mtl*, *zmdmp*, and *R1-nj* genes need to be fixed in the source germplasm. Moreover, inducers with purple-red kernels used as females in F_1_ crosses tend to negatively affect haploid identification after induction (**Figure 2**) because B1 Pl1 genes induce anthocyanin synthesis in the pericarp of purple corn (Petroni et al., 2014). In contrast, inducers with white/yellow kernels used as the female parent in F_1_ crosses enabled clear haploid identification (**Figure 2**), reducing efforts in haploid sorting and rogueing false-positive haploid plants in the haploid nursery.

### 4.3 Advanced improvement for inducers

The ability and efficiency to obtain DH lines from haploids influenced the utilization of DH technology. One major constraint is the proportion of haploid plants with restored male fertility after successful artificial genome doubling. QTL-enhancing haploid spontaneous genome doubling (SHGD) ability has been discovered (Chaikam et al., 2019a; Ren et al., 2020; Trampe et al., 2020; Verzegnazzi et al., 2021). Introgressing the desirable SHGD allele into inducer backgrounds could improve the proportion of haploid male fertility for obtaining more DH lines. Moreover, the high HIR performance of inducers increases the probability of haploids by self-induction, which would have a negative effect on the available pollen for haploid induction and line maintenance. Therefore, introgressing the desirable alleles from QTLs associated with SHGD into inducers is a target to further improve DHI line development by DH technology. Moreover, haploid inducibility of donors has been found to influence haploid induction rates (De La Fuente et al., 2018; Ren et al., 2022; Trampe et al., 2022; Trentin et al., 2022). Inadvertent selection likely increases haploid inducibility gradually when applying *in vivo* haploid induction for DHI line development over multiple selection cycles.

## Data availability statement

The data for this study can be found online: https://doi.org/10.25380/iastate.24527653.v1. The haploid inducers in this study could be accessed from Doubled Haploid Facility at Iowa State University.

## Author contributions

YC: Conceptualization, Data curation, Formal Analysis, Investigation, Visualization, Writing – original draft. TL: Funding acquisition, Project administration, Supervision, Writing – review & editing. UF: Conceptualization, Resources, Supervision, Validation, Writing – review & editing.
